# Physician and Nurse Well-Being and Preferred Interventions to Address Burnout in Hospital Practice: Factors Associated With Turnover, Outcomes, and Patient Safety

**DOI:** 10.1001/jamahealthforum.2023.1809

**Published:** 2023-07-07

**Authors:** Linda H. Aiken, Karen B. Lasater, Douglas M. Sloane, Colleen A. Pogue, Kathleen E. Fitzpatrick Rosenbaum, K. Jane Muir, Matthew D. McHugh

**Affiliations:** 1 Center for Health Outcomes and Policy Research, School of Nursing, and Leonard Davis Institute of Health Economics, University of Pennsylvania, Philadelphia

## Abstract

**Importance:**

Disruptions in the hospital clinical workforce threaten quality and safety of care and retention of health professionals. It is important to understand which interventions would be well received by clinicians to address the factors associated with turnover.

**Objectives:**

To determine well-being and turnover rates of physicians and nurses in hospital practice, and to identify actionable factors associated with adverse clinician outcomes, patient safety, and clinicians’ preferences for interventions.

**Design, Setting, and Participants:**

This was a cross-sectional multicenter survey study conducted in 2021 with 21 050 physicians and nurses at 60 nationally distributed US Magnet hospitals. Respondents described their mental health and well-being, associations between modifiable work environment factors and physician and nurse burnout, mental health, hospital staff turnover, and patient safety. Data were analyzed from February 21, 2022, to March 28, 2023.

**Main Outcomes and Measures:**

Clinician outcomes (burnout, job dissatisfaction, intent to leave, turnover), well-being (depression, anxiety, work-life balance, health), patient safety, resources and work environment adequacy, and clinicians’ preferences for interventions to improve their well-being.

**Results:**

The study sample comprised responses from 15 738 nurses (mean [SD] age, 38.4 [11.7] years; 10 887 (69%) women; 8404 [53%] White individuals) practicing in 60 hospitals, and 5312 physicians (mean [SD] age, 44.7 [12.0] years; 2362 [45%] men; 2768 [52%] White individuals) practicing in 53 of the same hospitals, with an average of 100 physicians and 262 nurses per hospital and an overall clinician response rate of 26%. High burnout was common among hospital physicians (32%) and nurses (47%). Nurse burnout was associated with higher turnover of both nurses and physicians. Many physicians (12%) and nurses (26%) rated their hospitals unfavorably on patient safety, reported having too few nurses (28% and 54%, respectively), reported having a poor work environment (20% and 34%, respectively), and lacked confidence in management (42% and 46%, respectively). Fewer than 10% of clinicians described their workplace as joyful. Both physicians and nurses rated management interventions to improve care delivery as more important to their mental health and well-being than interventions directed at improving clinicians’ mental health. Improving nurse staffing was ranked highest among interventions (87% of nurses and 45% of physicians).

**Conclusions and Relevance:**

This cross-sectional survey study of physicians and nurses practicing in US Magnet hospitals found that hospitals characterized as having too few nurses and unfavorable work environments had higher rates of clinician burnout, turnover, and unfavorable patient safety ratings. Clinicians wanted action by management to address insufficient nurse staffing, insufficient clinician control over workload, and poor work environments; they were less interested in wellness programs and resilience training.

## Key Points

**Question:**

Is clinician well-being a cause for concern, and if so, what interventions hold promise for retaining physicians and nurses in hospital practice?

**Findings:**

This cross-sectional multicenter survey study of 15 738 nurses and 5312 physicians found high and widespread burnout among clinicians in hospital practice that was associated with frequent turnover and patient safety concerns. In addition, clinicians lack confidence in management to resolve patient care problems and rated improvements in staffing and work environments as more important to their mental health and well-being than instituting clinician wellness and resilience programs.

**Meaning:**

These findings indicate that enhancing clinician well-being and retention requires deliberate actions by management to improve nurse staffing, work environments, and patient safety culture.

## Teaser

This cross-sectional survey study explores physician and nurse burnout, intent to leave, turnover, mental health, work-life balance, patient safety, and preferences for interventions at 60 US hospitals.

## Introduction

The hospital workforce remains in disarray despite the ebbing of the COVID-19 pandemic. The US Surgeon General has issued a national call to action in response to widespread reports that health care workers are reaching their breaking point.^1^ The US National Academy of Medicine is addressing the public health threat of high clinician burnout.^2^ Shortages of staff are among the top concerns of hospital leaders.^3^ Threats of strikes by physicians and nurses have increased, and progress in patient safety has slowed.^4,5^ The outpouring of public gratitude to clinicians during the height of the pandemic has failed to translate into actionable change by hospital management or public policies to address the causes associated with high clinician burnout and job dissatisfaction that predated and worsened during the COVID-19 pandemic.^6,7,8,9^

Knowledge of clinician well-being has mostly come from convenience samples of organizations and clinicians, and often from surveys of only physicians or only nurses.^9,10,11,12^ The US Clinician Wellbeing Study is a large, multisite collaborative investigation of the health and well-being of physicians and nurses during the COVID-19 pandemic practicing in 60 hospitals that received Magnet (American Nurses Credentialing Center) designation for being good places to work.^13^ This study explored the debate over whether interventions should prioritize bolstering the resilience of clinicians—a focus that angers many clinicians because it places the burden of adapting on them—or transforming hospital work environments to address modifiable sources of stress and burnout and to provide clinicians with more control over their work conditions.^14,15^

## Methods

This cross-sectional study was reviewed and deemed exempt by the institutional review boards of the University of Pennsylvania and of the participating hospitals. Prospective respondents received an invitation to participate and information on the study’s purpose and design, including its voluntary nature and the anonymity of responses. Completion of the survey represented informed consent. This study followed Strengthening the Reporting of Observational Studies in Epidemiology (STROBE) reporting guidelines for cross-sectional studies.

### Study Design, Data Collection, and Sample

This was a multisite collaborative study with data obtained via electronic surveys from more than 21 000 physicians (5312 respondents) and registered nurses (15 738 respondents) practicing in 60 US Magnet-recognized hospitals in 2021. The overall response rate was 26% (22% and 27% for physicians and nurses, respectively), and provided participating hospital-level information on turnover.

The Magnet Recognition Program is a voluntary institutional credentialing of good places to work based on nursing excellence and quality of health care as determined by the American Nurses Credentialing Center.^13,16^ Participating Magnet hospitals were enrolled as US *twinning* partners in the EU-funded Magnet4Europe intervention trial^17^ to improve mental health and well-being of clinicians in European hospitals.

In this study, the physician sample was drawn from hospitals with 10 or more physician-respondents and comprised 4064 attending physicians, 650 interns or residents, 212 fellows, and 125 other physicians from 53 hospitals. The registered nurse sample comprised 13 251 direct care nurses, 768 nurse managers, 194 advanced practice nurses, and 830 registered nurses in other positions from 60 hospitals.

Clinicians in adult medical and surgical specialties, including general inpatient units, intensive care units, and emergency departments, received electronic surveys. Each respondent described their mental health and well-being and the hospital’s staffing, management, patient safety, and quality of care. Respondents also ranked the importance of interventions to improve clinician well-being. Clinician surveys were modeled on multiple previous surveys of nurses from 1999 to 2021.^18^ Data collection occurred from January to June 2021. The final analytic sample included an average of 100 physicians and 262 nurses per hospital.

### Clinician Well-Being Measures

Burnout was measured using the 9-item Emotional Exhaustion subscale of the Maslach Burnout Inventory,^19,20^ which has been associated with patient outcomes.^21,22,23,24^ Respondents were classified as *high burnout *if their score was higher than the published top tertile for health care workers (≥27).^25^ This measure has been used extensively and validated with physicians and nurses.^26^ *Job dissatisfaction* and *intention to leave* their employer were measured by single-item questions.^27^ Staff turnover was provided by each hospital as the number of full-time equivalents (FTEs) who resigned, retired, or were terminated, divided by the number of actual FTEs during the same period (1 FTE is the equivalent of 2080 hours per year).

*Mental health* measures included anxiety, depression, and posttraumatic stress disorder (PTSD) associated with COVID-19, measured separately and together in a single measure. Anxiety was measured using Generalized Anxiety Disorder-2 item^28^ scale. *Depression* was measured using the Patient Health Questionnaire-2-item^29^ scale. Clinicians were classified as having a positive result of screening for anxiety or depression with a score of 3 or greater. *PTSD related to COVID-19* was determined using the Primary Care PTSD Screen for DSM-5.^30^ Clinicians were identified as having probable PTSD if they responded yes to 4 or more of the 5 questions regarding whether traumatic event associated with COVID-19 had affected them during the past month. Additional clinician well-being measures included single-item global self-assessments of *stress*, *work-life balance*,^31^ *overall health*, and *overall sleep*. Overall health used the global health rating item from the Short Form-8 Health Survey.^32^ Overall sleep quality was assessed using the global quality item from the Pittsburgh Sleep Quality Index.^33^

### Quality of Care and Patient Safety

*Quality of care* data provided by clinicians was in response to a single-item question that has been shown to be highly associated with mortality and other patient outcomes.^34^ *Patient safety grades* provided by clinicians ranged from A to F (per primary school grading system with no E grade available; unfavorable scores were C, D, and F).^35^ Clinicians rated *patient readiness to manage care after discharge* using a 4-point Likert scale dichotomized into “not confident” and “confident.” *Culture of patient safety* included 6 items from the Agency for Healthcare Research and Quality Hospital Survey on Patient Safety Culture V1 reported as separate items and as an average across all 6 items.^35^

### Resources and Management

*Staffing* was measured by asking clinicians whether there were enough nurses and to rate their own control over their workload.^36^ Items from the Practice Environment Scale of the Nursing Work Index^37,38^ were used to describe clinician relationships with hospital management and with team members. Clinicians were asked whether they would *recommend their hospital* as a good place to work or to friends and family in need of medical care.^39^ The *joyful workplace* measure was assessed using the Mini-Z 2.0,^40^ a validated tool used to determine workplace satisfaction and wellness. Clinicians were classified as having a “joyful” current workplace if their score on the Mini-Z was 40 or greater (range, 10-50).

### Interventions to Improve Clinician Well-Being

Clinicians were given a list of interventions generated from recommendations of the National Academy of Medicine^2^ and published research.^41,42^ Respondents selected the interventions that they thought would be most effective for alleviating burnout and improving clinician well-being.

### Statistical Analysis

Responses from individual physicians and nurses were aggregated within hospitals to determine how much clinician outcomes, measures of patient care quality and safety, staffing adequacy, and management measures ranged across hospitals, and these hospital means were then averaged across all hospitals to obtain an overall mean for each of the measures. Descriptive statistics (percentages, ranges) were calculated separately for physicians and nurses. Coefficients from multilevel models were obtained by regressing individual-level measures of the odds of physician and nurse burnout, job dissatisfaction, and intent to leave on grand median-centered hospital-level measures of resources and management and the safety and quality reports used. To evaluate the size and significance of these associations in a way that was consistent with other parts of this report, we converted the odds ratios resulting from these models to probabilities at the median of the predictor and multiplied the probabilities by 100 to express the results as differences in percentages:



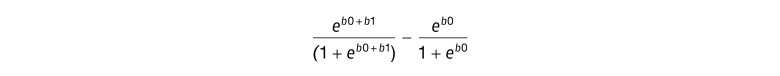



Because hospital turnover rates were provided at the hospital level, not the clinician level, ordinary least-squares regression models were used to estimate how physician and nurse turnover was affected by physician and nurse burnout, job dissatisfaction, and intent to leave. The coefficients from these regression models were further adjusted to indicate the differences in outcomes for physicians and nurses in hospitals at the 75th vs 25th percentiles of the different factors or independent variables or across similar ranges (eg, in hospitals at the 10th vs 60th percentile). We then described interventions that physicians and nurses reported as being the most and least important for improving their well-being.

Statistical tests were 2-tailed and *P* values less than .05 were considered statistically significant. Data analyses were performed from February 21, 2022, to March 28, 2023, using Stata, release 17 (StataCorp LLC).

### Sensitivity Analyses

Two sensitivity analyses were conducted to evaluate the robustness of our findings under different model specifications. Using ordinary least-squares regression models with all variables (on both sides of the equations) aggregated to the hospital level (eTable 1 in Supplement 1), we found that coefficients from the ordinary least-squares models estimating the associations of clinician outcomes with hospital resources, management, and patient safety were comparable with the coefficients derived from multilevel models in the main analysis. The second sensitivity analysis was conducted to determine whether the coefficients would be different if we excluded the 7 hospitals that had an insufficient number of physician responses. We present only comparisons of nurse coefficients in the 53 vs 60 hospitals because the physician coefficients would be unchanged. Using Hausman test, we found no meaningful differences in the results (eTable 2 in Supplement 1).

## Results

The total sample comprised the survey responses of 15 738 nurses practicing in 60 hospitals and 5312 physicians practicing in 53 of the same hospitals. Participating hospitals had an average of 100 physicians and 262 nurses, and the overall survey response rate was 26%. The nurse-respondents had a mean (SD) age of 38.4 (11.7) years; approximately 69% were female, 8% male, and 22% unknown/other sex; 11% were Asian, 4% Black, 5% Hispanic, 53% White, 4% other or multiracial, and 23% did not provide data. The physician-respondents had a mean (SD) age of 44.7 (12.0) years; approximately 35% were female, 45% male, and 21% unknown/other sex; 16% were Asian, 4% Hispanic, 5% other or multiracial, 52% White, and 21% did not provide data.

Across hospitals, an average of one-third of the physician-respondents and one-half of the nurse-respondents reported experiencing high burnout. However, the percentage with high burnout in both groups ranged substantially across hospitals, from 9% to 51% for physicians and 28% to 66% for nurses (Table 1). More than 1 of every 5 physicians (23%) reported that they would leave their current hospital within the year if possible, and the range across hospitals for physicians (6%-43%) suggests that in some hospitals as many as 30% to 40% would leave if they could. Over 40% of nurses would leave their current hospital if possible. Actual turnover reported by participating hospitals reveals an overall turnover rate for physicians of 6% and 17% for nurses. More than 4 in 10 physicians and 5 in 10 nurses report a great deal of stress because of their job, and in some hospitals those percentages were as high as 62% and 74%, respectively. Problems with overall health and sleep were more characteristic of nurses than of physicians, and decidedly more common in some hospitals than in others.

**Table 1.** Clinician Well-Being and Reports of Patient Safety and Quality of Care Across Hospitals

Measure	Mean (range) across hospitals, %^a^	
	Physicians in 53 hospitals	Nurses in 60 hospitals
**Survey respondents, No.**	5312	15 738
**Clinician well-being**		
High burnout	32 (9-51)	47 (28-66)
Job dissatisfaction	15 (0-33)	22 (2-48)
Intends to leave next year if possible	23 (6-43)	40 (21-69)
Turnover rate	6 (0-49)	17 (1-50)
Mental health		
(i) High anxiety	13 (0-25)	25 (10-37)
(ii) Likely depressed	9 (0-29)	17 (7-26)
(iii) Exhibits PTSD related to COVID-19	4 (0-15)	14 (3-27)
Morbidity includes i, ii, and/or iii	18 (0-33)	33 (18-48)
Great deal job-related stress	43 (11-62)	53 (35-74)
Work does not allow for personal/family life	32 (0-67)	18 (6-44)
Self-rated health is poor/fair	29 (6-75)	46 (29-62)
Self-rated quality of sleep is poor/fair	51 (25-83)	69 (50-88)
**Clinician reports of patient safety and quality of care**		
Patient safety grade, poor (C, D, or F^35^)	12 (0-40)	26 (3-63)
Quality of care is poor/fair	9 (0-30)	16 (2-55)
Not confident patients can manage postdischarge care	50 (25-78)	53 (31-72)
Summary measure of poor culture of patient safety, average score of 6	21 (12-31)	23 (10-31)
(i) Actions of management do not show that patient safety is a top priority	13 (0-26)	17 (3-45)
(ii) Clinicians feel mistakes are held against them	33 (16-58)	39 (11-50)
(iii) Patient information is lost during shift change when another is covering my patients	26 (6-45)	36 (19-52)
(iv) Do not feel free to question decisions or actions of authority	29 (9-48)	23 (3-41)
(v) Do not receive feedback about changes put into place based on event reports	19 (4-31)	16 (4-30)
(vi) Do not discuss ways to prevent errors from happening again	9 (0-27)	8 (0-19)

Abbreviation: PTSD, posttraumatic stress disorder.

^a^ Percentages are calculated at the hospital level, ie, the percentage of physicians with high burnout ranges from 9% in the hospital with the lowest percentage to 51% in the hospital with the highest percentage, and averages 32% across all hospitals. Poor culture of patient safety is the hospital average of 6 individual measures.

On patient safety, approximately 12% of physicians and 26% of nurses gave their hospital an unfavorable grade (C, D, or F), and for some hospitals more than one-quarter of the physician-respondents graded patient safety unfavorably. On average, 21% of physicians and 23% of nurses found fault with their hospitals’ culture of patient safety. One-third of physicians and 39% of nurses reported feeling their mistakes were held against them and 29% of physicians and 23% of nurses reported that they did not feel free to question authority. Although only 9% of physicians and 16% of nurses rated quality of care in their hospitals as poor or fair, in some hospitals those percentages rose to more than one-quarter of physicians and half of nurses. More than half of physicians and nurses across all hospitals and approximately three-quarters of both groups of clinicians in some hospitals were not confident that patients could manage their care after discharge.

Table 2 shows that 28% of physicians and more than half of nurses reported there were too few nurses. One-third of both physicians and nurses reported poor control over their workloads, and 39% of physicians and 63% of nurses reported a chaotic work environment. One of 5 physicians and one-third of nurses characterized the quality of their work environment as poor or fair. Approximately 42% of physicians and 47% of nurses reported lacking confidence that hospital management would resolve problems in patient care that clinicians identify, and close to one-third of physicians and half of nurses reported that the administration did not listen or respond to clinicians’ concerns. In some hospitals these percentages exceeded half of the clinicians. Less than 10% of clinicians described their workplace as joyful. Slightly more than 1 in 5 physicians and nurses reported not being involved in the internal governance of their hospital, and in some hospitals that problem was reported by approximately half of clinicians. Both groups of clinicians reported spending too much time on electronic health records (EHRs) and being frustrated by the task. On average across hospitals, close to 90% of physicians and nurses reported that professional relations between them were good and the majority reported that their care team worked efficiently together.

**Table 2.** Resources and Management Reported by Physicians and Nurses

Measure	Mean (range) across hospitals, %^a^	
	Physicians in 53 hospitals	Nurses in 60 hospitals
Survey respondents, No.	5312	15 738
Staffing		
Not enough nurses to care for patients	28 (0-57)	54 (22-93)
My control over my workload is poor or marginal	33 (13-51)	36 (19-63)
Overall quality of work environment		
Work environment is poor or fair	20 (0-44)	34 (8-64)
Work atmosphere is chaotic or tends to be chaotic	39 (19-63)	63 (36-86)
No clear philosophy of patient-centered care/nursing that pervades the clinical environment	15 (0-33)	20 (3-40)
Would not recommend hospital as a place to work	13 (0-42)	17 (1-57)
Would not recommend hospital to friends or family needing care	7 (0-22)	11 (0-35)
Joyful workplace	9 (0-30)	7 (0-20)
Management/clinician relations		
Not confident management will act to resolve problems in patient care that clinicians identify	42 (18-69)	47 (14-74)
Administration does not listen or respond to clinician concerns	29 (9-59)	47 (6-77)
Do not agree my values are well aligned with management	29 (0-48)	33 (9-57)
Clinicians are not involved in internal governance of hospital	23 (6-48)	22 (6-55)
Lack freedom to make important patient care and work decisions	14 (3-30)	24 (9-45)
Professional relations		
Physicians and nurses have good working relationship	94 (80-100)	89 (79-100)
Degree to which my care team works efficiently together is good/optimal	74 (53-93)	66 (54-87)
Electronic health records (EHRs)		
Time spent on EHRs is moderately high to excessive	74 (57-94)	57 (37-72)
EHRs adds frustration to daily work	62 (36-90)	44 (20-71)

^a^ Percentages are calculated at the hospital level, ie, the percentage of physicians who report that the work environment is “poor” or “fair” ranges from 0% in the hospital with the lowest percentage to 44% in the hospital with the highest percentage, and averages 20% across all hospitals.

Table 3 shows coefficients from multilevel models that regress hospital-level measures of physician and nurse outcomes (ie, burnout, job dissatisfaction, and intent to leave) on hospital-level measures of resources, management, and safety, after taking account of individual clinician differences in these outcomes. The hospital-level coefficients shown in Table 3 are percentage differences derived from odds ratios (described in the Statistical Analysis section previously) and indicate how different outcomes were for physicians and nurses in hospitals at the 75th rather than the 25th percentile of the independent variables (eg, resources). Hospitals characterized as having too few nurses, unfavorable work environments, and workloads that were beyond the control of clinicians had substantially more physicians and nurses who exhibited high burnout, job dissatisfaction, and intentions to leave their job. While the composite culture of patient safety scale appears to have little association with burnout of physicians, it does have a significant association with physician dissatisfaction and intent to leave and with all of the nurse outcomes. Significant effects shown in Table 3 are of substantive as well as statistical significance, given that they involve approximately 4% to 10% differences in job dissatisfaction and intent to leave for physicians in hospitals in which resource and management factors are at the 75th vs 25th percentile, and approximately 9% to 16% differences in all 3 outcomes for nurses.

**Table 3.** Coefficients From Multilevel Models Estimating the Differences in the Percentages of Clinicians With Various Outcomes (Burnout, Job Satisfaction, Intent to Leave) in Hospitals at the 75th vs 25th Percentiles of Resources, Management, and Patient Safety

Measure	Clinician outcomes					
	Physician coefficients, % (95% CIs)			Nurse coefficients, % (95% CIs)		
	Burnout	Job dissatisfaction	Intent to leave	Burnout	Job dissatisfaction	Intent to leave
Not enough nurses to care for patients (physician IQR, 16.8%-36.8%; nurse IQR, 40.6%-67.9%)	3.5 (0.2 to 7.1)^a^	4.8 (2.0 to 8.0)^b^	6.9 (4.1 to 9.9)^b^	11.5 (9.0 to 14.0)^b^	12.7 (10.3 to 15.3)^b^	16.2 (13.2 to 19.1)^b^
Control over workload is poor/marginal (physician IQR, 23.5%-40.6%; nurse IQR, 29.7%-42.2%)	10.1 (6.7 to 13.6)^b^	7.1 (3.2 to 11.5)^b^	8.9 (4.9 to 13.3)^b^	9.4 (7.3 to 11.6)^b^	10.8 (8.9 to 12.7)^b^	13.7 (11.3 to 16.2)^b^
Not confident that management will resolve problems (physician IQR, 33.3%-51.7%; nurse IQR, 40.2%-53.9%)	6.5 (3.3 to 9.8)^b^	6.6 (3.6 to 10.0)^b^	6.1 (2.8 to 9.6)^b^	9.3 (7.2 to 11.2)^b^	10.5 (8.6 to 12.3)^b^	11.7 (9.0 to 14.3)^b^
Work environment is poor/fair (physician IQR, 13.5%-26.4%; nurse IQR, 24.8%-40.8%)	6.7 (3.5 to 10.0)^b^	9.7 (7.6 to 12.2)^b^	10.7 (8.1 to 13.3)^b^	11.2 (9.3 to 13.1)^b^	12.2 (10.7 to 13.6)^b^	14.7 (12.3 to 17.2)^b^
Culture of patient safety average of 6 items^c^ (physician IQR, 18.6%-23.6%; nurse IQR, 19.5%-25.8%)	2.4 (−0.5 to 5.5)^d^	5.9 (3.3 to 8.9)^b^	5.4 (2.5 to 8.5)^b^	9.8 (6.9 to 12.7)^b^	11.5 (8.5 to 14.8)^b^	12.8 (9.1 to 16.6)^b^

^a^ *P *= .04

^b^ *P* < .001.

^c^ The 6 items in the summated *culture of patient safety* measure include (1) disagree patient safety is a priority, (2) agree that mistakes are held against staff, (3) agree that important information is lost during shift changes, (4) disagree that they feel free to question authority, (5) disagree that feedback about changes are put into place based on event reports, and (6) disagree that they discuss ways to prevent errors from happening again.

^d^ *P *= .11

Table 4 shows that both physician and nurse turnover were significantly associated with nurse burnout, nurse dissatisfaction, and nurses’ intentions to leave their current job. Physician turnover was approximately 4% to 5% higher and nurse turnover was 5% to 8% higher in hospitals in which nurse burnout rates, nurse job dissatisfaction rates, and the percentage of nurses that intended to leave were at the 75th vs the 25th percentile; physician burnout, dissatisfaction, and intent to leave were not associated with physician or nurse turnover.

**Table 4.** Coefficients Estimating the Differences in Clinician Turnover in Hospitals at the 75th vs 25th Percentiles for Burnout, Job Satisfaction, and Intent to Leave

Measure	IQR, %	Physician turnover		Nurse turnover	
		% (95% CI)	*P* value	% (95% CI)	*P* value
**Burnout rate**					
Physicians	28.8 to 37.0	0.7 (−2.6 to 3.9)	.69	0.6 (−3.1 to 4.2)	.76
Nurses	38.7 to 53.4	3.9 (0.1 to 7.7)	.046	5.1 (0.2 to 9.9)	.04
**Job dissatisfaction rate**					
Physicians	9.4 to 20.0	2.7 (1.7 to 7.1)	.23	3.9 (−1.5 to 9.2)	.16
Nurses	15.1 to 26.4	5.2 (1.8 to 8.6)	.004	5.4 (1.5 to 9.3)	.007
**Intent to leave rate**					
Physicians	17.0 to 28.4	0.9 (−4.1 to 5.9)	.73	3.7 (−2.2 to 9.5)	.22
Nurses	31.5 to 48.9	5.2 (1.1 to 9.2)	.013	8.4 (3.9 to 13.0)	< .001

Physicians and nurses were asked to select from a list of interventions those that they judged would be most effective in reducing burnout and improving their well-being. Responses from individual physicians and nurses were aggregated within hospitals as well as reported as percentages among all respondents across hospitals (Figure). The first 9 of the interventions were viewed as important by half or more of the nurses, including the 2 interventions that most nurses said would be important: improving nurse staffing levels and supporting clinicians in taking breaks without interruption. Physicians agreed that adequate nurse staffing and breaks without interruption were important to their well-being; they ranked reducing time spent on documentation and not having to routinely work unscheduled hours as “very important”; nurses agreed these were important to them as well. More resources to support new-to-practice clinicians were ranked highly by more than half of nurses and 39% of physicians. Poor EHR usability annoyed both physicians and nurses, and both groups wanted more individual control over scheduling. Half or more of physicians wanted reduced emphasis on meeting external quality metrics and clinician productivity targets. Notably, currently popular interventions adopted by management were not ranked as important by most clinicians, including clinician wellness champions, resilience training, and quiet places.

**Figure.**
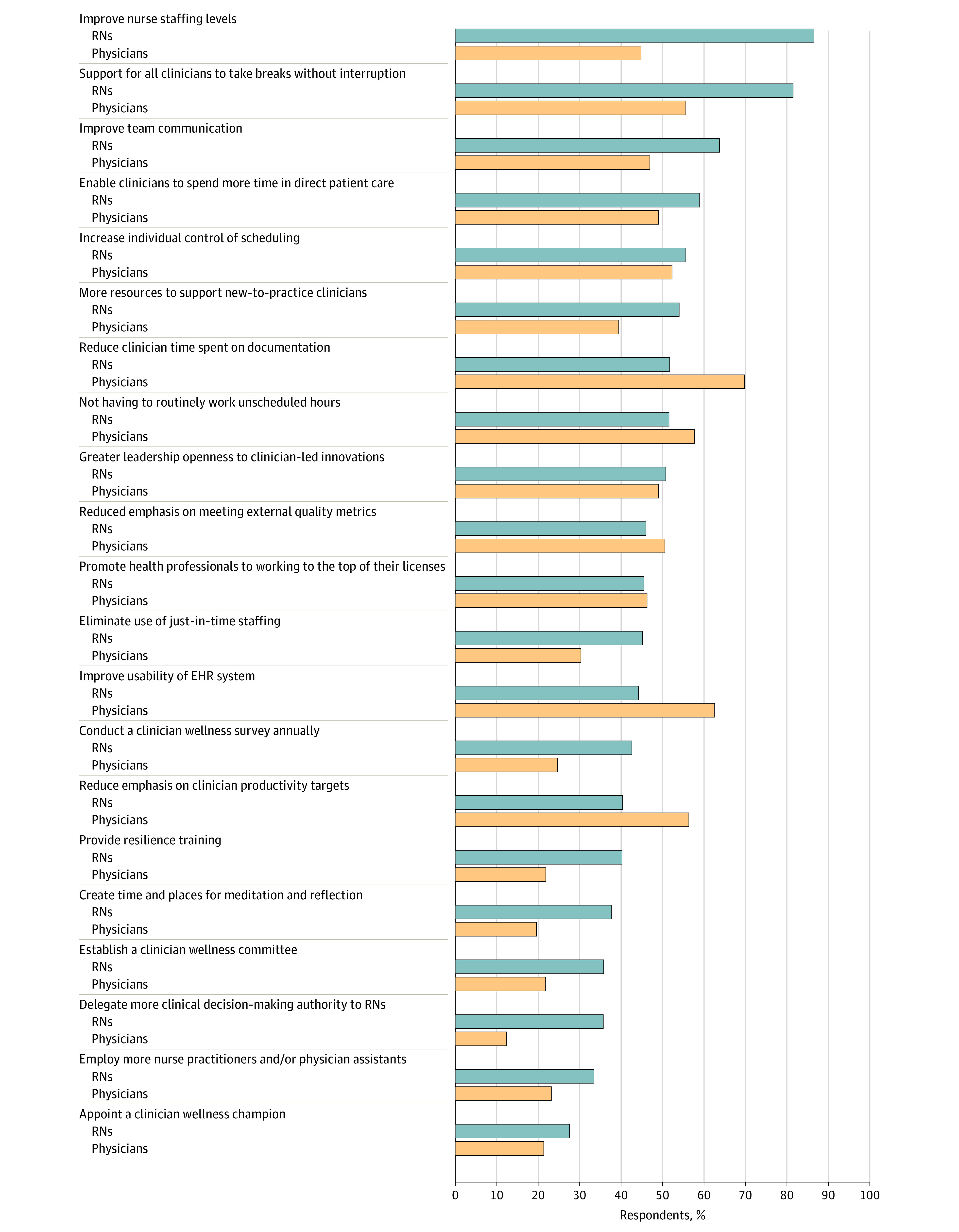
Interventions that Physicians and Nurses Ranked as “Very Important” to Improving Their Well-Being Percentages of physicians (yellow) and nurses in (blue) who responded “very important” to an intervention that could improve their well-being.

## Discussion

We provide detailed empirical evidence of substantial work-related health, mental health, and personal work-life balance challenges experienced by physicians and nurses in hospitals even at Magnet hospitals that have been formally recognized as being good places to work. Fewer than 10% of clinicians experienced joy in their hospital practices. Per our study findings, one-third of physicians and close to half of nurses are experiencing high burnout. One-third of physicians and one-half of nurses rate their own health as fair or poor. Physicians scored substantially worse than nurses on work-life balance, which was also a problem for 32% of physicians. Because the US Clinician Wellbeing Consortium is part of a 6-country European project to improve work environments,^17^ we found it notable that the US Accreditation Council on Graduate Medical Education partially addressed this problem in 2003 by limiting physician resident work hours to 80 per week while the EU limits physician resident work hours to 48 per week.

Hospitals that physicians and nurses characterized as having too few nurses, unfavorable work environments, and workloads that were beyond the control of clinicians had significantly more physicians and nurses that exhibited high burnout, job dissatisfaction, and intentions to leave their current job. For physicians, whether they have control over their workload was shown to be of paramount importance regarding level of burnout. For nurses, the factors of greatest importance to burnout were sufficiency of nurse staffing and quality of the work environment. Close to 90% of physicians and nurses reported that professional relations between them were good, and most reported that their care teams worked efficiently together. These findings hold promise for clinicians acting together to bring about important changes in their work environments. However, clinicians need management support for change, and our findings on clinician-management relations were concerning. Close to half of physicians and nurses were not confident that management would act to resolve problems that clinicians identify in patient care, and close to one-third of clinicians reported that their values were not well aligned with those of management. These are surprising findings in Magnet hospitals given that these issues may be even more pronounced in non-Magnet hospitals.^6^

The culture of patient safety is not usually discussed in the context of clinician well-being; however, our study found it to be significantly associated with physician dissatisfaction and intent to leave and with all nurse outcomes. The key tenets embraced in the culture of patient safety described 2 decades ago by the Institute of Medicine require close collaboration and trust between clinicians and management. This relationship seems to be lacking, as evidenced by the more than one-third of clinicians who reported that their errors were being held against them, and the 13% of physicians and 17% of nurses reporting that actions of management did not demonstrate that patient safety is an organizational priority.^43^

Both physicians and nurses prioritized interventions that influenced their ability to provide effective patient care over interventions focused on clinician wellness. Among their priority choices were improved nurse staffing (highly ranked by 45% of physicians and 87% of nurses) and improved work environments, including scheduled breaks without interruptions, not working unscheduled hours, more control over scheduling, and additional resources devoted to new-to-practice clinicians. Improving EHR usability and reducing emphasis on meeting external quality metrics were among the more highly ranked initiatives. Clinician wellness and resilience programs were ranked lowest, although they tended to be more commonly implemented than actions to improve clinicians’ working conditions. Research shows that physicians do not have a deficit in resilience but still experience job-related burnout, suggesting that other solutions are required.^14^

### Limitations

The study was conducted during the COVID-19 pandemic when clinician well-being was likely worse than previously, although research showed high rates of clinician burnout before the pandemic.^2,7^ Hospitals were Magnet-recognized and not representative of all hospitals (eTable 3 in Supplement 1). A similar simultaneous study of representative hospitals showed that nurse well-being and quality and safety assessments were significantly worse in non-Magnet hospitals.^6^ This study was cross-sectional, so caution is warranted in assuming causality. Lower response rates for email surveys are increasingly common and worsened during the pandemic.^44,45,46^ Our prior research^18^ using nurse surveys reveals no differences between respondents and resurveyed nonrespondents in the items studied. Other research surveying physicians have yielded lower response rates than this study.^14^ We used clinicians’ assessments of patient care quality and safety; previous research shows that clinician reports of patient quality are highly associated with independently measured patient outcomes.^34^

## Conclusions

This cross-sectional survey study found that physicians and nurses practicing in hospitals are under substantial stress, even in institutions known to be good places to work, which threatens the retention and vitality of the hospital workforce and patient safety. Clinicians report a lack of confidence in hospital management to act to resolve problems in patient care and to create supportive work environments and a work culture that promotes patient safety. Clinicians want improvements in nurse staffing and working conditions to address burnout and job dissatisfaction.

## Notes

Supplement 1. **eTable 1. **Coefficients from Ordinary Least Square Regression Models Estimating the Differences in the Percentages of Clinicians with Various Outcomes (Burnout, Job Satisfaction, Intent to Leave) in Hospitals at the 75th vs. 25th Percentiles of Resources, Management and Patient Safety **eTable 2.** Sensitivity Analysis Comparing Nurse Coefficients from 53 vs 60 Study Hospitals using Ordinary Least Square Regression Models to Estimate the Differences in the Percentages of Clinicians with Various Outcomes (Burnout, Job Satisfaction, Intent to Leave) in Hospitals at the 75th vs. 25th Percentiles of Resources, Management and Patient Safety **eTable 3.** Characteristics of Magnet and Non-Magnet Hospitals

Supplement 2. **Nonauthor Collaborators. **US Clinician Wellbeing Study Consortium

Supplement 3.
Data Sharing Statement
