# Supplementary material for: Physician and Nurse Well-Being and Preferred Interventions to Address Burnout in Hospital Practice: Factors Associated With Turnover, Outcomes, and Patient Safety
Source: JAMA Health Forum. 2023 Jul 7;4(7):e231809. doi: 10.1001/jamahealthforum.2023.1809 (PMC10329209; doi:10.1001/jamahealthforum.2023.1809)
Supplement: Supplement 1. — eTable 1. Coefficients from Ordinary Least Square Regression Models Estimating the Differences in the Percentages of Clinicians with Various Outcomes (Burnout, Job Satisfaction, Intent to Leave) in Hospitals at the 75th vs. 25th Percentiles of Resources, Management and Patient Safety eTable 2. Sensitivity Analysis Comparing Nurse Coefficients from 53 vs 60 Study Hospitals using Ordinary Least Square Regression Models to Estimate the Differences in the Percentages of Clinicians with Various Outcomes (Burnout, Job Satisfaction, Intent to Leave) in Hospitals at the 75th vs. 25th Percentiles of Resources, Management and Patient Safety eTable 3. Characteristics of Magnet and Non-Magnet Hospitals [file jamahealthforum-e231809-s001.pdf]

Supplemental Online Content

Aiken LH, Lasater KB, Sloane DM, et al; US Clinician Wellbeing Study Consortium. Physician and nurse well-being and preferred interventions to address burnout in hospital practice: factors associated with turnover, outcomes, and patient safety. *JAMA Health Forum*. 2023;4(7):e231809. doi:10.1001/jamahealthforum.2023.1809

eTable 1. Coefficients from Ordinary Least Square Regression Models Estimating the Differences in the Percentages of Clinicians with Various Outcomes (Burnout, Job Satisfaction, Intent to Leave) in Hospitals at the 75th vs. 25th Percentiles of Resources, Management and Patient Safety

eTable 2. Sensitivity Analysis Comparing Nurse Coefficients from 53 vs 60 Study Hospitals using Ordinary Least Square Regression Models to Estimate the Differences in the Percentages of Clinicians with Various Outcomes (Burnout, Job Satisfaction, Intent to Leave) in Hospitals at the 75th vs. 25th Percentiles of Resources, Management and Patient Safety

eTable 3. Characteristics of Magnet and Non-Magnet Hospitals

This supplemental material has been provided by the authors to give readers additional information about their work.

© 2023 Aiken LH et al. *JAMA Health Forum.*

eTable 1. Coefficients from Ordinary Least Square Regression Models Estimating the Differences in the Percentages of Clinicians with Various Outcomes (Burnout, Job Satisfaction, Intent to Leave) in Hospitals at the 75th vs. 25th Percentiles of Resources, Management and Patient Safety

	Clinician Outcomes					
	Physician Coefficients			Nurse Coefficients		
Resources, Management and Patient Safety	Burnout	Job Dissatisfaction	Intent to Leave	Burnout	Job Dissatisfaction	Intent to Leave
Not enough nurses to care for patients	3.7*	3.9*	4.4**	12.2***	12.2***	15.6***
(Physician IQR = 16.8% – 36.8%; RN IQR = 40.6% – 67.9%)	[0.3-7.1]	[0.9-6.9]	[1.4-7.4]	[9.7–14.8]	[10.0-14.4]	[12.8-18.4]
My control over my workload is poor or marginal	8.5***	4.6*	6.9***	9.8***	10.3***	13.0***
(Physician IQR = 23.5% – 40.6%; RN IQR = 29.7% – 42.2%)	[5.0-11.9]	[1.1-8.2]	[3.6-102]	[7.5-12.0]	[8.6-12.1]	[10.8-15.3]
Not confident management will act to resolve problems	6.4***	4.7***	4.3**	9.0***	9.2***	10.7***
(Physician IQR = 33.3% – 51.7%; RN IQR = 40.2% – 53.9%)	[3.5-9.3]	2.0-7.4]	[1.5-7.1]	[7.1-10.9]	[7.6-10.7]	[8.3-13.0]
Work environment is poor or fair	6.5***	6.8***	7.1***	11.4***	11.6***	14.0***
(Physician IQR = 13.5% – 26.4%; RN IQR = 24.8% – 40.8%)	[3.7-9.2]	[4.6-9.0]	[5.0-9.3]	[9.5-13.2]	[10.2-12.9]	[11.9-16.1]
Culture of Patient Safety Average (6 items) §	1.2	4.3***	3.7**	9.7***	9.9***	11.9***
(Physician IQR = 18.6% – 23.6%; RN IQR = 19.5% – 25.8%)	[1.6-4.0]	[2.1-6.5]	[1.4-6.0]	[6.9-12.5]	[7.4-12.4]	[8.5-15.3]

§ The 6 items in the summated Culture of Patient Safety Scale include 1) disagree patient safety is a priority, 2) agree that mistakes are held against staff, 3) agreed that important information is lost during shift changes, 4) disagree that they feel free to question authority, 5) disagree that feedback about changes are put into place based on event reports, and 6) disagree that they discuss ways to prevent errors from happening again.

© 2023 Aiken LH et al. *JAMA Health Forum.*

eTable 2. Sensitivity Analysis Comparing Nurse Coefficients from 53 vs 60 Study Hospitals using Ordinary Least Square Regression Models to Estimate the Differences in the Percentages of Clinicians with Various Outcomes (Burnout, Job Satisfaction, Intent to Leave) in Hospitals at the 75th vs. 25th Percentiles of Resources, Management and Patient Safety

	Number of Hospitals Used for Nurse Estimates					
	Burnout		Job Dissatisfaction		Intention To Leave	
IQR Change In -	53 Hospitals	60 Hospitals	53 Hospitals	60 Hospitals	53 Hospitals	60 Hospitals
Not enough nurses to care for patients	11.7*** [9.1-14.4]	12.2*** [9.7-14.8]	11.8*** [9.6-14.0]	12.2*** [10.0-14.4]	15.5*** [12.6-18.4]	15.6*** [12.8-18.4]
My control over my workload is poor or marginal	9.4*** [7.1-11.7]	9.8*** [7.5-12.0]	10.1*** [8.4-11.8]	10.3*** [8.6-12.1]	12.9*** [10.5-15.3]	13.0*** [10.8-15.3]
Not confident management will act to resolve problems	9.0*** [7.1-10.9]	8.9*** [7.1-10.8]	9.1*** [7.5-10.5]	9.2*** [7.6-10.7]	10.7*** [8.4-13.1]	10.7*** [8.3-13.0]
Work environment is poor or fair	11.4*** [9.6-13.2]	11.4*** [9.6-13.2]	11.3*** [9.9-12.6]	11.6*** [10.2-12.9]	14.0*** [11.8-16.2]	14.0*** [11.9-16.1]
Culture of Patient Safety Average (6 items) §	9.9*** [7.2-12.5]	9.7*** [6.9-12.2]	10.2*** [8.0-12.3]	9.9*** [7.4-12.4]	12.2*** [8.9-15.5]	11.9*** [8.5-15.3]

§ The 6 items in the summated Culture of Patient Safety Scale include 1) disagree patient safety is a priority, 2) agree that mistakes are held against staff, 3) agreed that important information is lost during shift changes, 4) disagree that they feel free to question authority, 5) disagree that feedback about changes are put into place based on event reports, and 6) disagree that they discuss ways to prevent errors from happening again.

© 2023 Aiken LH et al. *JAMA Health Forum.*

eTable 3. Characteristics of Magnet and Non-Magnet Hospitals

Hospital characteristics	Magnet Hospitals CWS (N = 60)	Non-Magnet Hospitals AHA (N = 4,429)
**Bed size, n (%)**		
<= 100	2 (3.33)	2,436 (55)
101-250	12 (20)	1,086 (24.52)
251-500	17 (28.33)	632 (14.27)
>500	29 (48.33)	275 (6.21)
**Ownership**		
Public	9 (15)	922 (20.82)
Non-profit	51 (85)	2,700 (60.96)
For-profit	0 (0)	614 (13.86)
Federal	0 (0)	193 (4.36)
**Location**		
Metro	58 (96.67)	2,616 (59.07)
Micro	2 (3.33)	758 (17.11)
Rural	0 (0)	1,055 (23.82)
**Region**		
New England	6 (10)	164 (3.7)
Mid Atlantic	21 (35)	348 (7.86)
South Atlantic	5 (8.33)	666 (15.04)
East North Central	14 (23.33)	678 (15.31)
East South Central	2 (3.33)	364 (8.22)
West North Central	2 (3.33)	655 (14.79)
West South Central	0 (0)	636 (14.36)
Mountain	2 (3.33)	412 (9.3)
Pacific	8 (13.33)	506 (11.42)
**Teaching status**		
Non-teaching	17 (28.33)	2,892 (65.3)
Minor teaching	21 (35)	1,211 (27.34)
Major teaching	22 (36.67)	326 (7.36)

Note. Minor teaching is defined by ≤ 1:4 medical residents or fellows to beds; Major teaching is defined by >1:4 medical residents or fellows to beds; Micropolitan is defined by >10,000 & 450,000 residents. Omnibus chi-square values (with multiple degrees of freedom) indicate that all hospital characteristics differ significantly in Magnet and Non-Magnet hospitals.

© 2023 Aiken LH et al. *JAMA Health Forum.*
