# Supplementary material for: Physician and Nurse Well-Being and Preferred Interventions to Address Burnout in Hospital Practice: Factors Associated With Turnover, Outcomes, and Patient Safety
Source: JAMA Health Forum. 2023 Jul 7;4(7):e231809. doi: 10.1001/jamahealthforum.2023.1809 (PMC10329209; doi:10.1001/jamahealthforum.2023.1809)
Supplement: Supplement 2. — Nonauthor Collaborators. US Clinician Wellbeing Study Consortium [file jamahealthforum-e231809-s002.pdf]

*Group Name(s): US Clinician Wellbeing Study Consortium							
*First Name and Middl	*Last Name	*Suffix (e	Academic Degrees	Institution	Location (city, state/province, country)	Role or Contribution	
Meagan	Cleary		PhD, BSN, RN	Advocate Christ Medical Center	Oak Lawn, IL/US	Site PI	
Cathaleen	Ley		PhD, RN	Anne Arundel Medical Center, Luminis Health	Annapolis, MD/US	Site PI	
Carla J.	Borchardt		DNP, RN	Avera McKennan Hospital & University Health Center	Sioux Falls, SD /US	Site PI	
Jeannine M.	Brant		PhD, APRN	Billings Clinic	Billings, MT /US	Site PI	
Barbra L	Turner		DNP, RN	Bon Secours Mercy Health/St. Elizabeth Youngstown Hospital	Youngstown, OH /US	Site PI	
Alyssa E.	Leimberger		MSN, RN	Bon Secours St. Mary's Hospital	Richmond, VA /US	Site PI	
Kristin	Kozlowski		MSN, RN	Bristol Health	Bristol, CT /US	Site PI	
Bernice L.	Coleman		PhD, RN	Cedars Sinai Medical Center	Los Angeles, CA /US	Site PI	
Nancy M.	Albert		PhD, RN	Cleveland Clinic Foundation	Cleveland, Oh /US	Site PI	
Caroline	Stewart		MSHI, RN	El Camino Health	Mountain View, CA /US	Site PI	
Dinah	Steele		DNP, RN	Emory Saint Joseph's Hospital	Atlanta, GA/US	Site PI	
Roberta	Kaplow		PhD, RN	Emory University Hospital	Atlanta, GA /US	Site PI	
Kathleen	Kaminsky		MS, RN	Englewood Health	Englewood, NJ /US	Site PI	
Heidi A.	Hinkle		MSN, RN	Good Samaritan	Vincennes, IN /US	Site PI	
Rocel D.	Besa		PhD, RN	Hackensack Meridian/Jersey Shore University Medical Center	Neptune, NJ /US	Site PI	
Kathleen P	Taylor		DNP, RN	Hackensack Meridian/Raritan Bay Medical Center	Perth Amboy, NJ /US	Site PI	
Kimberly	Dimino		DNP, RN	Hackensack Meridian/University Medical Center	Hackensack, NJ /US	Site PI	
Cecelia	Cetnar		MA, BSN, RN	Hackensack Meridian/Riverview Medical Center	Redbank, NJ /US	Site PI	
LS	Leach		PhD, RN	Huntington Health	Pasadena, CA /US	Site PI	
Sandra L.	Albritton		MN, BSN, RN	Kootenai Hospital	Coeur D Alene, ID /US	Site PI	
Carolyn L.	Davidson		PhD, RN	Lehigh Valley Hospital	Allentown, PA /US	Site PI	
Timothy	Carrigan		PhD, RN	Loyola University Medical Center	Maywood, IL /US	Site PI	
Debra A.	Burke		DNP, MBA	Massachusetts General Hospital	Boston, MA /US	Site PI	
Kristin R.	Anthony		MSN, RN	Mid Coast Hospital	Brunswick, ME /US	Site PI	
Mildred O.	Kowalski		PhD, RN	Morristown Medical Center	Morristown, NJ /US	Site PI	
Martha	Rounds		MS, APRN	Newport Hospital	Newport, RI /US	Site PI	
Jennifer M.	Tudor		MSN, RN	Northbay Healthcare	Fairfield, CA/ US	Site PI	
Leigh	Griffis		DNP, RN	Northwell Health/Huntington Hospital	Huntington, NY / US	Site PI	
Linda M.	Vassallo		MSN, RN	Northwell Health/Long Island Jewish Medical Center	New Hyde Park, NY / US	Site PI	
Marie	Mulligan		PhD, RN	Northwell Health/Mather Hospital	Port Jefferson, NY / US	Site PI	
Irene	Macyk		PhD, RN	Northwell Health/North Shore Medical Center	Manhasset, NY / US	Site PI	
Catherine	Manley-Cullen		MS, RN	Northwell Health/Northern Westchester Hospital	Mount Kisco, NY / US	Site PI	
Sandra L.	Hutchinson		MSN, RN	Northwestern Medicine Delnor Hospital	Geneva, IL/ US	Site PI	
Amanda E.	Haberman		MSN, RN	Northwestern Medicine/Central DuPage Hospital	Winfield, IL / US	Site PI	
Amy L.	Barnard		MS, APRN	Northwestern Medicine/Lake Forest Hospital	Lake Forest, IL / US	Site PI	
Barbara H.	Gobel		MS, RN	Northwestern Memorial Hospital	Chicago, Il / US	Site PI	
Diana L.	McMahon		DNP, RN	Ohio State University Comprehensive Cancer Center - The James	Columbus, OH / US	Site PI	
Megan J.	Brown		MSN, RN	OSF HealthCare Saint Anthony Medical Center	Rockford, IL / US	Site PI	
Lisa	Strack		MSN, RN	OSF Healthcare St. Joseph Medical Center	Bloomington, IL / US	Site PI	
Sheryl A.	Emmerling		PhD, RN	OSF HealthCare/Saint Francis Medical Center	Peoria, IL / US	Site PI	
Angela R.	Coladonato		DNP, RN	Penn Medicine/Chester County Hospital	West Chester, PA / US	Site PI	
Jessie A.	Reich		PhD, RN	Penn Medicine/Hospital of the University of Pennsylvania	Philadelphia, PA / US	Site PI	
Justin J.	Gavaghan		BSN, RN	Penn Medicine/Lancaster General Hospital	Lancaster, PA / US	Site PI	
James R.	Ballinghoff		DNP, MBA, RN	Penn Medicine/Penn Presbyterian Medical Center	Philadelphia, PA/ US	Site PI	
Florence D.	Vanek		MSN, RN	Penn Medicine/Pennsylvania Hospital	Philadelphia, PA/ US	Site PI	

*First Name and Middl	*Last Name	*Suffix (e	Academic Degrees	Institution	Location (city, state/province, country)	Role or Contribution	
Karyn A.	Book		DNP (c), RN	Penn Medicine/Princeton Health	Plainsboro, NJ / US	Site PI	
Kathy	Easter		MSN, RN	Robert Wood Johnson University Hospital	New Brunswick, NJ/ US	Site PI	
Pamela	Duchene		PhD, APRN	Southwestern Vermont Health Care	Bennington, VT/ US	Site PI	
Mary E.	Lough		PhD, RN	Stanford Health Care	Stanford, CA / US	Site PI	
Christine L.	Benson		MSN, RN	Summa Health System Akron Campus	Akron, OH/ US	Site PI	
Maria	Ducharme		DNP, RN	The Miriam Hospital	Providence, RI / US	Site PI	
Paul	Quinn		PhD, RN	The Valley Hospital	Ridgewood, NJ/ US	Site PI	
Donna M.	Molyneaux		PhD, RN	Thomas Jefferson University Hospital and Gwynedd Mercy University, G	Philadelphia and Gwynedd Valley, PA / US	Site PI	
Lori	Kennedy		PhD, RN	UC Davis Health	Sacramento, CA / US	Site PI	
Elizabeth Ellen	Nyheim		MSN, RN	UC San Diego Health	San Diego, CA / US	Site PI	
Donna M.	Grochow		MSN, RN	UCI Health	Orange, CA / US	Site PI	
Shannon M.	Purcell		DNP, RN	University of Alabama at Birmingham Hospital	Birmingham, AL/ US	Site PI	
Kirsten	Hanrahan		DNP	University of Iowa Health Care	Iowa City, IA/ US	Site PI	
Kathy B.	Isaacs		PhD	UK HealthCare	Lexington, KY/ US	Site PI	
Jill J.	Whade		MSN, RN	WakeMed Health and Hospitals	Raleigh, NC/US	Site PI	
