# Supplementary material for: Physician and Nurse Well-Being and Preferred Interventions to Address Burnout in Hospital Practice: Factors Associated With Turnover, Outcomes, and Patient Safety
Source: JAMA Health Forum. 2023 Jul 7;4(7):e231809. doi: 10.1001/jamahealthforum.2023.1809 (PMC10329209; doi:10.1001/jamahealthforum.2023.1809)
Supplement: Supplement 3. — Data Sharing Statement [file jamahealthforum-e231809-s003.pdf]

Data Sharing Statement

Aiken. Physician and Nurse Well-Being and Preferred Interventions to Address Burnout in Hospital Practice. *JAMA Health Forum*. Published July 07, 2023. doi:10.1001/jamahealthforum.2023.1809

Data
Data available: No
